# Identifying Health Systems Gaps as Perceived by Postpartum Patient Navigators at an Urban Academic Medical Center

**DOI:** 10.1177/26884844251362313

**Published:** 2025-07-23

**Authors:** Hannah M. Green, Maya Daiter, Viridiana Carmona-Barrera, Laura Diaz, Brittney Williams, Ka’Derricka Davis, Joe Feinglass, Arjeme Cavens, Charlotte Nizik, Brigid M. Dolan, Michelle A. Kominiarek, William A. Grobman, Lynn M. Yee

**Affiliations:** ^1^ Division of Maternal-Fetal Medicine, Department of Obstetrics and Gynecology, Northwestern University Feinberg School of Medicine, Chicago, Illinois, USA.; ^2^ Division of General Internal Medicine, Department of Medicine, Northwestern University Feinberg School of Medicine, Chicago, Illinois, USA.; ^3^ Division of General Obstetrics and Gynecology, Department of Obstetrics and Gynecology, Northwestern University Feinberg School of Medicine, Chicago, Illinois, USA.; ^4^ Department of Medical Education, Northwestern University Feinberg School of Medicine, Chicago, Illinois, USA.; ^5^ Division of Maternal-Fetal Medicine, Department of Obstetrics and Gynecology, Brown University, Providence, Rhode Island, USA.

**Keywords:** patient navigation, postpartum care, systems improvement, care transition

## Abstract

**Background:**

Although the postpartum period is an opportunity to promote long-term well-being and health systems usage, system complexities limit patients’ abilities to optimize their longitudinal health. Postpartum patient navigation, an intervention that assists individuals in navigating health systems, is a novel innovation that may mitigate barriers to longitudinal care.

**Methods:**

Within a recently completed randomized controlled trial (RCT), we conducted a secondary analysis of interviews with two navigators and a subset (*N* = 15) of navigated participants to describe gaps in the health care system. Semi-structured interview guides were used to conduct 11 sets of interviews with postpartum navigators. In the RCT, navigators supported publicly insured individuals for 1 year postpartum. Interviews focused on relationships with patients and care teams and reflections on health systems gaps which challenged care. Interviews were transcribed and analyzed using grounded theory. A randomly selected subset of interviews with navigated participants was analyzed to triangulate navigator-identified gaps.

**Results:**

Navigators identified three major gaps in the care system for postpartum individuals: overall health system challenges, postpartum care challenges, and gaps in the transition to primary care. Health system challenges included fragmentation within the hospital system, fragmentation across distinct health care institutions, high task burden for patients, and lack of clear communication between patients and care teams. Postpartum care challenges included operational and logistical errors in care and the transient nature of obstetric care. Gaps in the transition to primary care included a lack of emphasis on the importance of primary care, lack of administrative support in the transition, and lack of communication between care teams.

**Conclusion:**

Postpartum patient navigators elucidated health systems gaps that present challenges in maximizing the longitudinal well-being of birthing individuals. These results identify areas for systems improvements that could promote lifelong health.

## Introduction 

Although the peripartum period is one of increased access to care and thus a unique opportunity to improve the long-term health of birthing individuals, it is characterized by marked socioeconomic and racial disparities in care utilization and health outcomes.^1–5^ These disparities persist throughout the postpartum period, in which less than 60% of publicly insured individuals attend a postpartum visit within 8 weeks of delivery.^6^ Thereafter, care shifts to the purview of a primary care physician, yet a significant portion of birthing individuals do not attend a primary care appointment within 1 year, with lower rates among Black and publicly insured individuals.^7^ The complexity and fragmentation of the American health system complicate this care transition for patients, especially those with chronic illnesses or pregnancy-related complications who may require additional non-obstetric specialty care.^8,9^ Given that pregnancy and the postpartum period are opportunities to improve birthing individuals’ long-term well-being,^10,11^ ensuring postpartum individuals transition to longitudinal care is paramount to improving overall, not just obstetric, health outcomes.

Patient navigation, an individualized intervention in which a trained individual assists patients in addressing barriers to care and navigating the health system, has been implemented in a variety of specialties^12–14^ and is emerging as a promising intervention to reduce postpartum health disparities.^15,16^ As a multilevel and interdisciplinary intervention that interfaces with patients, providers, and health systems administrators,^17^ patient navigation offers a uniquely comprehensive lens to view the postpartum care landscape and subsequent transition to long-term care. Multiple studies have assessed systems-level factors in obstetric postpartum care utilization,^18^ yet literature assessing postpartum individuals’ experiences with the broader health system has largely focused on individual-level determinants of care utilization.^19–23^ There is a growing interest in assessing health care at a systems level for postpartum individuals, with particular attention to the transition to primary care.^24–26^

This study aimed to describe existing systems-level gaps in postpartum care for low-income individuals, as identified through postpartum patient navigators. Understanding these gaps may help to guide future program development and policy interventions both within and beyond obstetrics to improve care and health outcomes for postpartum individuals as they transition to longitudinal care.

## Materials and Methods

This is a qualitative analysis of interview data collected within a recently concluded randomized controlled trial (RCT) of postpartum patient navigation for low-income individuals receiving Medicaid-funded prenatal care. The RCT, Navigating New Motherhood 2 (NNM2) (NCT03922334),^27^ randomized 405 pregnant and postpartum individuals over 4 years to receive either usual care or a patient navigation intervention. Patients randomized to patient navigation received individualized navigation services for up to 13 months after delivery from a non-health professional trained as a patient navigator.^28^ Two trained navigators participated in NNM2, and a substitute navigator provided temporary coverage for one navigator’s brief leave. Both main navigators identified as Hispanic women and were bilingual in English and Spanish. Participants were assigned a single navigator who provided continuous services over the course of the intervention. The primary aim of the RCT was to evaluate whether postpartum patient navigation improves clinical outcomes as compared with usual care; trial analysis is ongoing, and no data from the overarching trial are presented herein.

As part of the NNM2 protocol, we developed a semi-structured interview guide to conduct serial interviews with patient navigators, focusing on navigators’ relationships with patients, relationships with care teams, and health systems gaps that challenged care. Each of the main navigators participated in 11 and 10 interviews, respectively, while the substitute navigator participated in a single interview, yielding 22 total interviews. The first eight sets of interviews were conducted approximately every 3 months over the first 2 years of the RCT, and the remaining interviews were conducted every 6 months for the remainder of the trial. Interviews lasted approximately 1 hour and were conducted by the primary author, who had no prior relationship with the navigators. Navigators did not receive compensation for their participation aside from routine employment-based benefits.

Navigator interviews were audio-recorded, transcribed, and uploaded to Dedoose (www.dedoose.com), a qualitative data management and analysis software, and analyzed using the constant comparative method. The initial set of interviews yielded a primary codebook, which was iteratively refined and finalized with the entire NNM2. This finalized codebook was then applied to subsequent interviews. For participant checking, navigators themselves participated in the codebook creation process, but all coding and analysis were completed by the primary author. Review of transcripts was not blinded. This article focuses on thematic analysis of data specifically discussing navigator-identified gaps in the health system.

To triangulate these data, interviews with NNM2 participants randomized to receive postpartum navigation were also analyzed. As part of the NNM2 study protocol, a randomly selected group of 50 participants (40.0% Hispanic or Latina, 53.3% African American; mean age 28.4 [standard deviation 4.78] years) receiving the intervention participated in two 30-minute interviews with research staff. The first interview was conducted *via* telephone at 3–6 months postpartum, and the second in person at 11–13 months postpartum upon study exit. These interviews aimed to understand participants’ experiences with navigation and perceptions of the program. As part of the informed consent process, participants were aware their interviews would be analyzed for multiple purposes, including for sub-analyses in combination with other data sources. Participants received a $50 remuneration for their participation in interviews.

For this analysis, 15 sets of participant interviews were randomly selected, transcribed, and analyzed using Dedoose qualitative data software. Given that each participant completed 2 interviews, a total of 30 interviews were analyzed. This number was chosen *a priori* and, after analysis, was deemed sufficient after all NNM2 team members agreed saturation had been reached. After reviewing the first 10 transcripts, two researchers, including the primary author, created a codebook for analysis, which was finalized with the entire team and applied to the remaining transcripts. Each researcher coded transcripts independently, and disagreements were discussed until concordance was reached. The primary author then used the themes identified in navigator interviews to sort participant interview data and applied these themes to identify concordant quotations for analysis. To ensure themes were represented by more than one navigator, themes were only considered if they were confirmed by two or more navigators. Additionally, to ensure the navigator-identified themes were triangulated with patient experiences broadly, rather than individual narratives, these themes were corroborated with interviews from all 15 participants.

The NNM2 study was approved by the Northwestern University Institutional Review Board. The investigators received verbal consent from navigators at the start of each interview, as well as signed consent from all enrolled participants.

## Results

Qualitative analysis of 22 interviews with patient navigators revealed that they perceived gaps in the care system for postpartum individuals across three major themes: overall health system challenges, postpartum care challenges, and gaps in the transition to primary care (Table 1). Subthemes across these domains were identified. Analysis of interviews with navigated participants corroborated both overarching themes and subthemes (Table 2). Themes and subthemes are discussed in depth below.

**Table 1.** Navigator-Identified Gaps in the Postpartum Care System

Theme	Subtheme	Exemplary quotation
Overall health system challenges	Fragmentation within hospital system	“I have a patient [with] diabetes . . . She has a lot of appointments. . . . every time she went to see the doctor, she came out with five other appointments . . . I wasn’t sure if . . . [the care teams] were communicating amongst themselves . . . I felt like there were some gaps and . . . some overlap.”
	Fragmentation across institutions	“Someone needed an X-ray image . . . that they got at [an outside institution] . . . She’d been waiting on that in order to . . . get surgery . . . I called her orthopedic surgeon’s office . . . and let them know the situation . . . She’s tried calling, I’ve tried calling, nothing’s working . . . maybe a provider-to-provider request would expedite things. So, I asked if a note can be made . . . to get this going.”
	High task burden for patients	“The doctor’s telling you you need a breast pump, but is the doctor helping you fill out the prescription form? If you missed a call, is the doctor reaching out . . . to ask if they could please call you again?”
	Lack of clear communication between patient and care teams	“She kept getting . . . really high . . . blood glucose readings . . . I reached out to the endocrinologist, and I said, ‘ . . . My patient is under the assumption that you’re monitoring her Dexcom . . . she’s getting a lot of high glucose readings and nobody’s reaching out to her . . . ’ [The endocrinologist] explained . . . ‘We don’t monitor her Dexcom . . . she should call the clinic and let them know that it’s an emergent hyperglycemia . . . ’”
Postpartum care challenges	Operational and logistical errors in care	“A patient came in for a mood check . . . usually done two to three weeks . . . postpartum . . . The doctor did a full examination and [noted] . . . it was a six week . . . visit . . . so there was no further follow up, but she was only three weeks postpartum . . . The patient was unaware . . . like, ‘Oh, I’m done with my appointment . . . ’ If I weren’t her patient navigator, then this would [have] been it for her, even though she’d still had . . . more healing to do.”
	Transient nature of obstetric care	“After they have their . . . six-week postpartum appointment, they’re told by the doctor, ‘ . . . you should follow up with your primary care doctor in six to twelve months.’ So, unless something comes up, they’re not really doing anything in regards to their health.”
Gaps in the transition to primary care	Lack of emphasis on importance of primary care	“Many patients don’t know that primary care is important. [They ask], ‘So I can set up with a doctor, and I can just always go back to them?’ Like, yeah! Even if you don’t need them . . . ”
	Lack of administrative support in transition	“I genuinely feel that some patients would not have . . . established primary care . . . One of my patients . . . [said], ‘Oh yeah, thanks for helping me connect with the doctor because I didn’t even know how to do it and I would have been like ‘Forget it.’ . . . To me it doesn’t seem that complex like just call and make an appointment, right? But for individuals who are . . . really busy or . . . get asked questions about insurance, all that can seem very complex . . . ”
	Lack of communication between care teams	“One of my patients transitioned over to primary care . . . she did have GDM when she was pregnant, [so] I wanted to make sure she was following up with the recommendations given to her by the doctor during her six-week postpartum appointment. And when I reached out to the primary care clinic, I felt like there was so much disconnect in what they knew and . . . didn’t know.”

**Table 2.** Patient Perceptions of Navigator-Identified Gaps in the Postpartum Care System

Theme	Subtheme	Exemplary quotation
Overall health system challenges	Fragmentation within hospital system	“I did need multiple doctor’s appointments…I see different physicians so she did talk to the different people for me and stuff like that”
	Fragmentation across institutions	“[Navigation was most helpful] getting all my paperwork from my old doctor’s office to my new doctor’s office”
	High task burden for patients	“ . . . she’s very . . . helpful . . . after having a baby I have been going through like a lot . . . I couldn’t . . . handle it by myself . . . she was helping me [with] a lot of stuff like planning doctor’s appointments . . . I was trying to do everything for a while on my own, but it was like nobody was available for a long time. She helped me . . . [There’s] a lot of frustrating stuff that you have to go through . . . She was really helpful.”
	Lack of clear communication between patient and care teams	“ . . . [Navigators] establish . . . that link between a patient who doesn’t understand what is happening in the medical dynamic . . . and doctors who don’t have the time . . . to devote that space to us . . . there is an intermediate point where there is a gap, if [navigation] didn’t exist.”
Postpartum care challenges	Operational and logistical errors in care	“ . . . I went to claim . . . the pills I take for depression, and they didn’t want to give them to me . . . The doctor’s signature was not authorized to prescribe that time of medicine. [My navigator] helped to renew the authorization the very same day and they were able to give me the medicine.”
	Transient nature of obstetric care	“[In obstetrics], everything was bounced around, you’d always see somebody new . . . And it’s better to keep with the same person because they’re kind of more familiar with your health.”
Transition to primary care	Lack of emphasis on importance of primary care	“Like even regarding follow-up . . . I’m very proactive when it comes to my healthcare but I don’t think I would have made this [primary care] appointment . . . her reminding me and saying, ‘Hey establish care,’ that was helpful”
	Lack of administrative support in transition	“At no time was I ever offered this extra assistance . . . Now having it be my fourth pregnancy, and finally getting a primary care . . . it took for me to get to my fourth pregnancy before I was able to get to someone . . . who was able to assist me”
	Lack of communication between care teams	“ . . . it was just getting the right information to me for me to comprehend exactly what I needed to do [to transition my care]”

### Overall health system challenges

Navigators identified overall health system features that challenged patients’ ability to access and receive quality health care throughout the postpartum year. Subthemes included fragmentation within the hospital system, fragmentation across distinct health care institutions, high task burden for patients, and lack of clear communication between patients and care teams (Table 1). Participant quotations corroborated these subthemes (Table 2).

#### Fragmentation within the hospital system

Navigators identified various challenges in patients’ ability to move through the health care system within a single institution. Primarily, they noted that needing referrals for distinct specialists posed a challenge to care: “…the healthcare system is so complex . . . it . . . delays patient care … there was a patient who . . . received a referral to a program and because she didn’t engage [immediately], even though the referral said it expired next year, [the specialists] were like, ‘Oh her [primary care provider] needs to put in another referral…’” Such challenges were exacerbated when patients required appointments with multiple specialists. One navigator, for example, recounted the process of connecting a patient to care to prepare for bariatric surgery, which required coordinating referrals and appointment assistance for providers in five distinct departments.

This complexity, navigators noted, challenged patients’ ability to even begin the process of connecting with providers, as it was often unclear which department was most appropriate for patient care. Navigators recounted multiple instances of interfacing with obstetric staff to identify whether a patient’s health concern was appropriate for the obstetrician/gynecologist team, primary care provider (PCP), or subspecialist. Patients corroborated this challenge, stating, “I don’t know who to call…” and citing navigators as “connecting me with the right office.” Even when patients could contact clinic representatives themselves, navigators stated that patients “have to wait through pressing prompts…leave a message with the patient service representative [who]…routes the message to the doctor or the nurse…it’s easier for patients to reach out to us.” In patient interviews, participants agreed, stating the “simplicity to have access [to the care system *via* the navigator] is very beneficial.”

Siloed departments within the institution also challenged patient care. For example, one navigator recalled a patient who was told she needed preoperative medical clearance, but “the scheduler [told me], ‘Oh yeah, we changed that’…The departments, depending on who you talk to…give you different information…” Disjointed insurance coverage similarly challenged care. Navigators recounted multiple patients who received referrals to primary or specialty care within the same institution in which they received their prenatal care, only to find their insurance was not accepted by that department.

#### Fragmentation across distinct health care institutions

Additional systems challenges emerged as navigators coordinated care across various health care institutions. Transferring medical records between institutions and disparate electronic health records required significant navigator involvement (Table 1), and patients cited this task as one of the most helpful navigation services (Table 2). One navigator recalled telling a patient, “‘Because your [primary] care is not with [our institution]…you should go to your primary care doctor [for]…a referral…to see a specialist here.’ I had to keep reaching out to the PCP…calling, leaving a voicemail…follow[ing] up…which…resulted…in a delay in the patient’s [specialty] care.” Patients, too, described how navigators frequently contacted their providers at outside institutions for care coordination: “she would call them on my behalf…I don’t think I would have been able to do that.”

#### High task burden for patients

Receiving quality postpartum care required patients themselves to complete an abundance of tasks. Navigators recalled that, consequently, patients faced time constraints preventing them from obtaining care: “if the patient only has 15 minutes to make an appointment…she’s on hold for 10…when they finally talk to her they’re like, ‘We’re going to take a message…’ And then when the nurse calls them [back] they could very well be at work and not answer.” These time constraints were exacerbated by the transition to parenthood: “…small things like [scheduling appointments]…can go a long way. ‘Cause that means 5 extra minutes with…their baby…Taking care of themselves.” Ensuring insurance coverage and scheduling transportation similarly challenged patients. In interviews, patients stated that task coordination was a prominent barrier to care and required significant time and energy to navigate often-confusing institutions. One patient stated, “I’ve got these kids…I don’t have [the time]…Sometimes it’s like a three-ring circus going on in here…” Patients repeatedly noted that they didn’t think they would have accomplished nearly as many health-related tasks without navigator assistance (Table 2).

This high task burden was a cognitive load for patients; one stated, “I do lose track of a whole lot because I’m trying to remember everybody else’s stuff including my own.” Patients described the mental workload of remembering the ‘postpartum to-do list’: “…when you have kids, it’s all about them. We always forget about us…because [you’re focused on] everybody else.” Navigators recalled spending significant time reminding patients of appointments, following up on tasks that patients completed, and supporting patients in understanding the importance of these tasks.

#### Lack of clear communication between patients and care teams

Finally, navigators reported that a lack of clear communication between patients and their care teams inhibited high-quality care. These communication challenges occurred within obstetric, primary, and subspecialty care (Table 1). Patients often identified navigators as a useful “in-between communicator” who ensured there was “open communication” between them and their care team, especially regarding confusing health topics. One patient noted, “Even just like vocabulary [was helpful]. . . .” Other patients recalled multiple conversations with navigators to ensure patients’ health comprehension. Navigators agreed, citing gaps in communication between patients and their care teams. One navigator, for example, recalled a patient who told her physician she understood the risks and benefits of a medication, “And then [she told me] ‘I don’t know what this is for. How is it going to affect me?’”

Similarly, navigators recalled patients facing challenges communicating their personal priorities for their care, largely due to limited self-advocacy and knowledge. One navigator recalled, “Some people . . . they’re vocal, and they advocate for themselves . . . but I haven’t found that to be the majority by far.” Patients corroborated these challenges, stating they did not realize some concerns could be shared with their care team, but their navigator “wrote a list of things for me [to discuss]…And she made sure the doctor addressed it.”

### Postpartum care challenges

Specific to postpartum care, navigators identified operational and logistical errors, as well as the transient nature of obstetric care, as significant challenges to streamlined care (Table 1). Patients corroborated these subthemes (Table 2).

#### Operational and logistical errors in care

Navigators recalled numerous instances in which they brought attention to and ameliorated operational errors in postpartum care provision. These errors often involved inaccurate appointment scheduling, including scheduling patients for the incorrect appointment (Table 1) or scheduling patients as new, rather than returning, patients, leading to extensive delays. Initially, for example, clinic protocol required that existing patients with short-interval pregnancies be scheduled as new patients. Navigators reported that this “delay[ed] their care…[it] makes no sense that you’re starting them fresh…They’re already an established patient…”

Miscommunications between team members sometimes led to appointment-related issues, as well. One navigator recalled a patient who required a follow-up colposcopy, stating, “The nurses were under the impression that they should have received a message from the doctor…but the doctor forgot or put her on the wrong list…So if I would not have brought this up…the nurses were not going to [schedule her].” Patients were often unaware of these appointment-related errors, which were realized by navigators and addressed prior to affecting patient care. In the absence of a navigator, however, these errors could have delayed or negatively impacted care provision. Certain issues, however, such as problems with medication prescriptions, were realized by patients and delayed receipt of care (Table 2).

Overall health system fragmentation exacerbated these logistical errors. One patient, for example, “told the doctor she already had [a vaccination at an external institution]. So then the doctor just wrote off that the patient received it…[When I contacted the outside institution], the nurses confirmed that she didn’t have it. . . .” This example highlights how overall health system challenges compounded challenges specific to postpartum care provision.

#### Transient nature of obstetric care

Obstetric care typically ceases after the postpartum visit, usually between 6 and 12 weeks after delivery. Navigators identified this transient nature of obstetric care as a challenge. They recalled, for example, hesitance from postpartum clinic nurses or social workers to engage in longitudinal patient care: “…they say like, ‘Well, this is their last visit…we’re not gonna see them anymore…this is really short-term care…six weeks and then they’re done with us.’” Patients, too, corroborated how the transient nature of obstetric care affected their experience and limited their ability to longitudinally engage with providers (Table 2).

Such time-limited care also affected patients’ perceptions of their need for continued health care. Navigators noted that, given that obstetric care is framed as short-term care, “…once they’re done with postpartum, they feel like they’re done with their healthcare. Like, ‘Oh okay that’s it…I don’t have to go to the doctor anymore until something comes up.’” Despite this sentiment, patients often had numerous outstanding health issues requiring non-obstetric care, which may not have been addressed without navigator support for engaging in longitudinal care.

### Gaps in the transition to primary care

After postpartum care concluded, patient care transitioned to primary and specialty care. Navigators identified numerous gaps in this transition including a lack of emphasis on the importance of primary care, a lack of administrative support in the transition, and a lack of communication between care teams (Table 1), all of which were corroborated by patients (Table 2).

#### Lack of emphasis on the importance of primary care

When discussing efforts to facilitate patients’ connection to a PCP, navigators noted that many patients did not understand the importance of primary care, a consequence of inadequate emphasis on its importance by the obstetric team (Table 1). Navigators noted that “Not everybody gets a referral [to a PCP]” from postpartum providers, even if they do not have an established primary provider. The lack of focus on the transition to primary care during the postpartum visit made care continuity less salient for patients. As such, navigators recalled frequently “reminding patients” about the utility of primary care. Patients corroborated this, noting, “I don’t think I would have made [a primary care appointment] without patient navigation.”

Furthermore, navigators reported that this limited emphasis on the importance and role of a PCP led to unrealistic expectations of the health system. One navigator described a patient “ask[ing for] an appointment for tomorrow” for nonurgent, albeit bothersome, symptoms. Similarly, navigators recalled patients recurrently visiting the emergency department for concerns appropriate for a PCP. One navigator explained, “We can use that to educate…why it’s important for you to have a PCP and be able to stay connected.”

#### Lack of administrative support in transition

Both navigators and patients also noted a lack of administrative support regarding the transition from obstetric to primary care. Navigators reported that patients felt overwhelmed by the process of transitioning to primary care, and some patients would not have connected to a PCP without navigator support (Table 1). Patients agreed, consistently citing navigators’ assistance with the primary care transition as one of the most helpful parts of the navigation program. Inconsistent insurance coverage, an example of health system fragmentation that is exacerbated for postpartum patients with Medicaid, posed additional challenges for patients. One navigator noted, “…The patient will leave the appointment thinking, ‘I got a referral [to a PCP] and . . . I have to break the news…’you can’t come here [because of your insurance].’ And they’re like, ‘But why? They gave me the referral.’”

To ameliorate these administrative challenges, navigators recalled working closely with patients to coordinate insurance-covered PCP appointments and ensure a smooth transition of care: “I have a [high-risk] patient who … had to follow up with a PCP … And it always felt like she was too busy. So we strategized … Let’s come up with ways for her to actually show up for her appointments and make appointments.” Patients agreed, stating navigators “went out of [their] way to make sure that I got a primary care physician…” and connected to long-term care.

#### Lack of communication between care teams

Even after patients were able to connect with a PCP, navigators identified gaps in communication between the obstetric and primary care teams. Pregnancy-related information that was pertinent to a patient’s continued care often did not reach the primary care team (Table 1). Consequently, navigators reported frequently interfacing directly with the PCP, providing a bridge of information between postpartum and primary care: “…they were super grateful [for the information]. They were like, ‘These are some of the things we address. Do you not want us to discuss it?’ Like, for example, birth control. They’re like ‘We address birth control, but is that something they’re doing with their OB/GYN doctor?’” These stories clearly identified gaps in communication between postpartum and primary care teams.

## Discussion

This qualitative analysis utilized data from a recently concluded RCT of postpartum patient navigation to identify gaps in the health care system for postpartum individuals. Trained patient navigators identified health system gaps across three main themes: overall health system challenges, postpartum care challenges, and gaps in the transition to primary care. Within overall health systems features, navigators identified fragmentation both within and across institutions, high patient task burden, and lack of clear communication between patients and care teams as challenges to streamlined patient care. Within postpartum care specifically, operational errors and the transient nature of obstetric care threatened high-quality care provision. Finally, as patients transitioned to primary care, navigators noted a lack of emphasis on the importance of primary care, a lack of administrative support, and a lack of communication between care teams. Interviews with navigated participants corroborated navigator-identified themes and subthemes.

**FIG. 1.**
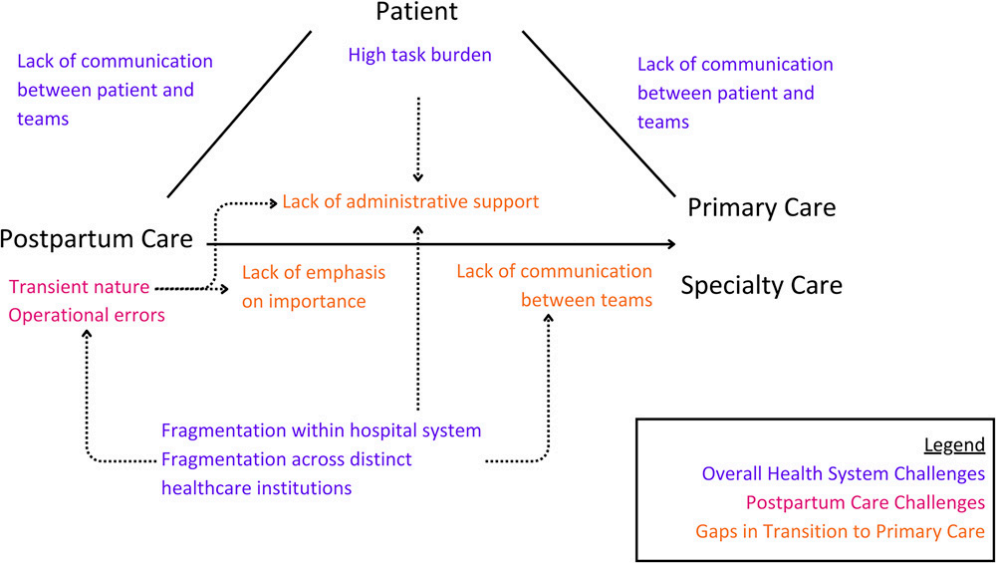
Navigator-identified gaps throughout the postpartum care system. Dotted arrows represent interconnected themes.

Importantly, these navigator-identified gaps were interconnected (Fig. 1). Fragmentation within and across institutions, for example, led to operational errors in care and exacerbated a lack of communication between obstetric and primary care teams. Distinct electronic medical records between institutions compounded these communication challenges. Likewise, disjointed insurance policies added additional administrative tasks for patients and complicated the transition from postpartum to longitudinal care. Without patient navigation, patients must navigate these complexities independently, generating a high task burden for patients within a health system that lacks intrinsic support for the transition from postpartum to long-term care. This challenge is exacerbated by the transient nature of obstetric care and limited emphasis by obstetric providers on the importance of primary care. For patients with comorbidities or pregnancy-related complications, the need for specialty care further complicates this transition.

The postpartum landscape is a complex period of care, as patients navigate care transitions, novel health issues requiring specialty follow-up, and anticipatory guidance for future health, all the while balancing the transition to parenthood. Existing literature corroborates many of the navigator-identified gaps elucidated in this analysis, including communication challenges, lack of care continuity, and patient difficulty navigating fragmented care.^29–31^ This analysis, however, uses patient navigation, a multilevel, comprehensive, and interdepartmental care intervention, to identify health systems gaps, and thus offers a uniquely holistic picture of the postpartum care system.

This study suggests that systems modifications are required to optimize both postpartum and long-term care after pregnancy. If shown to be evidence-based, patient navigation offers a unique opportunity to address systems gaps given its comprehensive, individualized approach to care. Navigation is a resource-intensive intervention, however, and simple systems improvements could also help to optimize this complicated landscape (Table 3). Improved patient portals for care management, for example, could alleviate a high task burden for patients, ameliorate a lack of administrative support in the transition to primary care, and improve communication between care teams. Primary care transition plans created during the postpartum visit could both emphasize the importance of primary care and facilitate communication between obstetric and primary care teams, drawing explicit attention to pregnancy complications that carry long-term health implications. Simplified appointment scheduling processes would ameliorate a high patient task burden, limit operational errors, and improve patient support for care transitions. Health policy interventions to simplify insurance coverage could also streamline care. This study thus provides targeted areas for new interventions, at the provider, health systems, and policy levels, which could improve the care landscape for postpartum individuals.

**Table 3.** Interventions or Systems Improvements to Address Navigator-Identified Gaps

Theme	Subtheme	Possible interventions to address gap
Overall health system challenges	Fragmentation within hospital system	- Streamlined specialty referral processes - Improved systems of interdepartmental communication - Physical consolidation of care
	Fragmentation across institutions	- Improved interconnectedness between electronic medical records - Streamlined medical records transfer process - Initiatives to improve health systems literacy
	High task burden for patients	- Simplified appointment scheduling processes - Improved patient health portals for care management and appointment scheduling
	Lack of clear communication between patient and care teams	- Patient education initiatives - Improved post-visit instructions - Increased interpretation services
Postpartum care challenges	Operational and logistical errors in care	- Simplified appointment scheduling processes - Improvements in electronic medical record to catch operational errors
	Transient nature of obstetric care	- Emphasis on longitudinal well-being during obstetric care - Improved handoff between obstetric and primary care - Co-location of obstetric and primary care
Transition to primary care	Lack of emphasis on importance of primary care	- Emphasis on longitudinal well-being during obstetric care - Initiatives to improve health systems literacy
	Lack of administrative support in transition	- Improved handoff between obstetric and primary care - Improved patient health portals for care management - Simplified appointment scheduling processes
	Lack of communication between care teams	- Improved systems of interdepartmental communication - Improved handoff between obstetric and primary care - Improved patient health portals for care management

This analysis has a few limitations to note. Navigation occurred within a single large urban health care system within the context of an RCT. The complex nature of our tertiary care center, a multi-facility hospital system with a variety of subspecialists, could have created or exacerbated systems challenges that may not be present in other care systems. This, paired with our small sample size of three trained navigators, may limit our findings’ generalizability to other health systems. Reassuringly, however, our findings are similar to those identified in existing literature conducted within a variety of health systems.^21,25,32,33^ Additionally, the perceptions of patient navigators may be biased, as individuals are likely to more saliently identify areas in which their role is useful, and thus their perceptions may overstate systems gaps. Triangulation with patient data, however, suggests that this bias did not account for navigators’ perceptions. This analysis does not address gaps external to the care system, including social barriers to care or state-specific Medicaid policy, both of which significantly impact the postpartum care landscape and warrant inclusion in program development and systems improvements. Finally, it is well known that both postpartum and primary care providers have significant time constraints, resource limitations, and competing priorities during patient visits,^34,35^ all of which likely contribute to the gaps identified in this analysis. Future work should investigate how these time and resource constraints create system gaps and widen health disparities.

## Conclusions

Postpartum patient navigation is a novel intervention to address health disparities among birthing individuals and offers a unique lens through which to view the care system. Our analysis of navigator-identified gaps in the care system highlights opportunities for systems improvement at multiple levels. While patient navigation represents one method to address these gaps, additional initiatives, including primary care transition programs and systems modifications to reduce health care fragmentation, are likely required to improve the postpartum care landscape and thus the long-term health of birthing individuals.

## Data Sharing and Availability

Due to the sensitive nature of qualitative data that may reveal participant identity, individual data cannot be shared. Qualified researchers who meet criteria for access to qualitative data may contact the corresponding author/authors and receive contact information for the institutional review board.

## Abbreviations Used

NNM2: Navigating New Motherhood 2
RCT: randomized controlled trial

## References

[1] Bullington BW, Arora KS. Fulfillment of desired postpartum permanent contraception: A health disparities issue. Reprod Sci 2022;29(9):2620–2624; doi: 10.1007/s43032-022-00912-3 35713848 PMC10120182

[2] Gadson A, Akpovi E, Mehta PK. Exploring the social determinants of racial/ethnic disparities in prenatal care utilization and maternal outcome. Semin Perinatol 2017;41(5):308–317; doi: 10.1053/j.semperi.2017.04.008 28625554

[3] Mi T, Hung P, Li X, et al. Racial and ethnic disparities in postpartum care in the greater Boston area during the COVID-19 pandemic. JAMA Netw Open 2022;5(6):e2216355; doi: 10.1001/jamanetworkopen.2022.16355 35737390 PMC9226999

[4] Phibbs CM, Kristensen-Cabrera A, Kozhimannil KB, et al. Racial/ethnic disparities in costs, length of stay, and severity of severe maternal morbidity. Am J Obstet Gynecol MFM 2023;5(5):100917; doi: 10.1016/j.ajogmf.2023.100917 36882126 PMC10121928

[5] Yee LM, Simon MA, Grobman WA, et al. Pregnancy as a “golden opportunity” for patient activation and engagement. Am J Obstet Gynecol 2021;224(1):116–118. 32979375 10.1016/j.ajog.2020.09.024

[6] Tenfelde S, Joyce C, Tell D, et al. Reducing disparities in postpartum care utilization: Development of a clinical risk assessment tool. J Midwifery Womens Health 2023;68(2):179–186; doi: 10.1016/j.ajog.2020.09.024 36565235 PMC10089952

[7] Essien UR, Molina RL, Lasser KE. Strengthening the postpartum transition of care to address racial disparities in maternal health. J Natl Med Assoc 2019;111(4):349–351; doi: 10.1016/j.jnma.2018.10.016 30503575

[8] Ruderman RS, Dahl EC, Williams BR, et al. Provider perspectives on barriers and facilitators to postpartum care for low-income individuals. Womens Health Rep (New Rochelle) 2021;2(1):254–262; doi: 10.1089/whr.2021.0009 34318295 PMC8310741

[9] Theilen LH. Pregnancy as a window to future health: What next? BJOG 2020;127(12):1498; doi: 10.1111/1471-0528.16354 32511833

[10] Dassanayake M, Langen E, Davis MB. Pregnancy complications as a window to future cardiovascular disease. Cardiol Rev 2020;28(1):14–19; doi: 10.1097/CRD.0000000000000253 31008769

[11] Gilmore LA, Klempel-Donchenko M, Redman LM. Pregnancy as a window to future health: Excessive gestational weight gain and obesity. Semin Perinatol 2015;39(4):296–303; doi: 10.1053/j.semperi.2015.05.009 26096078 PMC4516569

[12] Cervantes L, Hasnain-Wynia R, Steiner JF, et al. Patient navigation: Addressing social challenges in dialysis patients. Am J Kidney Dis 2020;76(1):121–129; doi: 10.1053/j.ajkd.2019.06.007 31515136 PMC8118353

[13] Rohloff M, Peifer G, Thompson JH. Patient navigation for overactive bladder improves access to care. Int Urogynecol J 2020;31(5):1007–1012; doi: 10.1007/s00192-019-04085-7 31463529

[14] Roland KB, Higa DH, Leighton CA, et al. Client perspectives and experiences with HIV patient navigation in the United States: A qualitative meta-synthesis. Health Promot Pract 2020;21(1):25–36; doi: 10.1177/1524839919875727 31597497 PMC6917848

[15] Brown Z, Messaoudi C, Silvia E, et al. Postpartum navigation decreases severe maternal morbidity most among Black women. Am J Obstet Gynecol 2023;229(2):160 e1–e8; doi: 10.1016/j.ajog.2023.01.002 36610531

[16] McKenney KM, Martinez NG, Yee LM. Patient navigation across the spectrum of women’s health care in the United States. Am J Obstet Gynecol 2018;218(3):280–286; doi: 10.1016/j.ajog.2017.08.009 28844825 PMC5826769

[17] Green HM, Carmona-Barrera V, Diaz L, et al. Implementation of postpartum navigation for low-income individuals at an urban academic medical center. PLoS One 2023;18(2):e0282048; doi: 10.1371/journal.pone.0282048 36821597 PMC9949671

[18] Wouk K, Morgan I, Johnson J, et al. A systematic review of patient-, provider-, and health system-level predictors of postpartum health care use by people of color and low-income and/or uninsured populations in the United States. J Womens Health (Larchmt) 2021;30(8):1127–1159; doi: 10.1089/jwh.2020.8738 33175652 PMC8403215

[19] Gemkow JW, Liss DT, Yang T-Y, et al. Predicting postpartum transition to primary care in community health centers. Am J Prev Med 2022;63(5):689–699; doi: 10.1016/j.amepre.2022.05.010 35840450 PMC10228376

[20] Bennett WL, Chang H-Y, Levine DM, et al. Utilization of primary and obstetric care after medically complicated pregnancies: An analysis of medical claims data. J Gen Intern Med 2014;29(4):636–645; doi: 10.1007/s11606-013-2744-2 24474651 PMC3965743

[21] Shankar M, Chan CS, Frayne SM, et al. Postpartum transition of care: Racial/Ethnic Gaps in Veterans’ Re-Engagement in VA Primary care after pregnancy. Womens Health Issues 2021;31(6):603–609; doi: 10.1016/j.whi.2021.06.003 34229932

[22] Paez KA, Eggleston EM, Griffey SJ, et al. Understanding why some women with a history of gestational diabetes do not get tested for diabetes. Womens Health Issues 2014;24(4):e373–e379; doi: 10.1016/j.whi.2014.04.008 24981396

[23] Interrante JD, Admon LK, Carroll C, et al. Association of health insurance, geography, and race and ethnicity with disparities in receipt of recommended postpartum care in the US. JAMA Health Forum 2022;3(10):e223292; doi: 10.1001/jamahealthforum.2022.3292 36239954 PMC9568809

[24] Cameron NA, Birdsell H, Niznik CM, et al. An enhanced postpartum transition program to primary care. J Womens Health (Larchmt) 2024;33(10):1417–1422; doi: 10.1089/jwh.2023.0465 38634543 PMC12698300

[25] Clapp MA, Ray A, Liang P, et al. Postpartum primary care engagement using default scheduling and tailored messaging: A randomized clinical trial. JAMA Netw Open 2024;7(7):e2422500; doi: 10.1001/jamanetworkopen.2024.22500 39012630 PMC11252898

[26] Geissler KH, Jeung C, Attanasio LB. Preventive primary care in the postpartum year: The role of medicaid delivery system reform. Am J Prev Med 2024;67(2):184–192; doi: 10.1016/j.amepre.2024.03.005 38484901 PMC11260532

[27] Yee LM. Navigating New Motherhood 2 (NNM2) clinicaltrials.gov. National Insititutes of Health US National Library of Medicine; 2024. Available from: https://clinicaltrials.gov/study/NCT03922334?term=navigtaing%20new%20motherhood&rank=1

[28] Yee LM, Williams B, Green HM, et al. Bridging the postpartum gap: Best practices for training of obstetrical patient navigators. Am J Obstet Gynecol 2021;225(2):138–152; doi: 10.1016/j.ajog.2021.03.038 33812809 PMC8328879

[29] Harrell T, Howell EA, Balbierz A, et al. Improving postpartum care: Identifying opportunities to reduce postpartum emergency room visits among publicly-insured women of color. Matern Child Health J 2022;26(4):913–922; doi: 10.1007/s10995-021-03282-5 34982328 PMC8724640

[30] Kraus AC, Quist-Nelson J, Ryan S, et al. Postpartum care in a cardio-obstetric clinic after preterm preeclampsia: Patient and healthcare provider perspectives. Am J Obstet Gynecol MFM 2024;6(5):101339; doi: 10.1016/j.ajogmf.2024.101339 38492641

[31] Shellhaas C, Conrey E, Crane D, et al. The Ohio gestational diabetes postpartum care learning collaborative: Development of a quality improvement initiative to improve systems of care for women. Matern Child Health J 2016;20(Suppl 1):71–80; doi: 10.1007/s10995-016-2170-2 27502198 PMC6697553

[32] Phillips SEK, Celi AC, Wehbe A, et al. Mobilizing the fourth trimester to improve population health: Interventions for postpartum transitions of care. Am J Obstet Gynecol 2023;229(1):33–38; doi: 10.1016/j.ajog.2022.12.309 36574875

[33] Strelow B, Herndon J, Ferrier A, et al. Identifying discrepancies in gestational diabetes mellitus postpartum follow-up during care transitions. J Prim Care Community Health 2023;14:21501319231214072; doi: 10.1177/21501319231214072 38041430 PMC10693797

[34] Krishnamurti T, Simhan HN, Borrero S. Competing demands in postpartum care: A national survey of U.S. providers’ priorities and practice. BMC Health Serv Res 2020;20(1):284; doi: 10.1186/s12913-020-05144-2 32252757 PMC7137294

[35] Nguyen MT, Honcharov V, Ballard D, et al. Primary care physicians’ experiences with and adaptations to time constraints. JAMA Netw Open 2024;7(4):e248827; doi: 10.1001/jamanetworkopen.2024.8827 38687477 PMC11061766

