# Supplementary material for: Identifying Health Systems Gaps as Perceived by Postpartum Patient Navigators at an Urban Academic Medical Center
Source: Womens Health Rep (New Rochelle). 2025 Jul 23;6(1):691–701. doi: 10.1177/26884844251362313 (PMC12413253; doi:10.1177/26884844251362313)
Supplement: Supplementary Data S2 [file 26884844251362313_supp_datas2.docx]

Research Question: What is the role of a patient navigator in providing care for low-income postpartum women? How did patients benefit from patient navigators?

Introduction

Thank you for your participation in this interview as we aim to gain feedback about your experiences being a patient navigator. You are being asked to participate because you are a patient navigator for the Navigating New Motherhood study. In order to get your feedback, we are going to ask some general questions regarding your role, your experiences with your patients, and your experiences with the care team. We will not ask you any specific questions about you or your health.

Before we begin, I just want to remind you that there are no wrong answers. Please feel free to provide honest and open feedback about your experiences with the navigation program. We are trying to get a better understanding of your experiences during this time so that the program can be improved in the future. Our conversation will be recorded to make sure I do not miss anything. You can choose to stop participating at any time. Do you have any questions?

Interview Questions

**General Questions**

- Now that you have more experience as a patient navigator, how would you define your role?
- How do you feel your navigation strategies have evolved as you gain more experience?
  - Can you tell me about a specific instance you used one of these strategies?
- How has your average day as a patient navigator changed as patients move later into the postpartum period?
- How do you document your experiences as a patient navigator and the issues facing your patients?

**Relationship with Patients**

- What are some of the effective ways you have developed a relationship with patients?
  - Could you recount a time where you felt you made strides in developing a relationship with a patient in the last three months?
- What strategies do you use to build trust with your patients?
  - Can you share an example of a time you developed trust with a patient in the last three months?
- How have your relationships changed with patients who are now later in the postpartum period?
  - Can you tell me an example of a relationship you have with a patient that has shifted in this way?
- What are the biggest challenges to maintaining relationships with patients?
  - Can you tell me a specific example in which you found it challenging to maintain a relationship with a patient in the last three months?
- How have patients’ needs shifted as they move later into the postpartum period?
  - Can you share an example of a patient whose needs have shifted later into the postpartum period?
  - How have you adapted to this shift?
- How do you prepare patients for accessing healthcare after the navigation relationship ends?
  - Can you share a time where you felt you prepared patients for accessing care after the navigation period?

**Relationship with Care Team**

- How do you feel about your integration into patients’ postpartum care team as patients move later into the postpartum period?
  - Can you share a time in the last three months you felt integrated into patient care?
  - Can you share a time in the last three months you felt like an outsider in the patient care team?
- How does your role as a patient navigator connect the patient to their care team as patients move later into the postpartum period?
- How has your communication with the patient’s care team evolved as patients move later into the postpartum period?
  - Can you share an example from the last three months which illustrates this shift?
- How would you describe the relationship between yourself, the patient, and the patient’s physician as patients move later into the postpartum period?
  - Can you tell me a specific example of interactions between yourself, the patient and the physician that occurred within the last three months which illustrate this relationship?
- How would you describe your relationship with patients’ nurses?
  - Can you tell me a specific example of an interaction that occurred within the last three months which illustrates this relationship?
- In what instances have you felt you needed to direct a patient to a social worker, rather than assist them yourself?
  - Can you share a specific example of a time in the last three months you connected a patient to a social worker?
- How has your relationship with patients’ non-obstetric care providers changed as patients move later into the postpartum period?
  - Can you share an example within the last three months of an interaction you had with a patient’s non-obstetric care team?
- What are the biggest challenges you face in working with patients’ care team?
  - Can you tell me about a specific time in the last three months you experienced this challenge?

**Gaps in the Healthcare System**

- What barriers to accessing care have you witnessed your patients experience later on in their postpartum care? Examples may include transportation issues, lack of health literacy, unstable housing, affordability etc.
  - Can you share a time in the last three months a patient faced a barrier to accessing her care?
  - How does your role as a navigator help patients to overcome these barriers?
- What challenges do you witness patients experience later in their postpartum care? Examples of challenges may include insurance issues, issues with appointments, lack of health literacy, miscommunication with physicians etc.
  - Could you tell me a specific story in the last three months where a patient experienced challenges in her healthcare?
  - How do you feel navigation addresses these challenges?

**Longevity of Navigation**

- How do you think hospital systems or obstetric clinics could benefit from a patient navigation program?
  - Can you share a time where you felt your role improved the clinical system?
- What types of hospital systems or patient populations do you believe would benefit most from a patient navigation program?
- How can directors of patient navigation programs promote the development of successful and sustainable navigators both prior to beginning the role and throughout?
- How do you feel the close level of involvement with patients affects the sustainability of patient navigation? Have you experienced any challenges in your role due to the emotional burden or workload intensity of being a navigator? What resources or support would have helped with this?
- What do you think the biggest challenges are to implementing a postpartum patient navigation program?
- What advice would you have for future patient navigators?

**Personal Reflection**

- What do you like most about being a patient navigator?
- Where do you feel your time is most useful as a patient navigator?
  - Can you tell me about a time in the last three months you felt useful in a patient’s postpartum care?
- Where do you feel you have spent time that hasn’t been useful for patients?
  - Can you tell me about a time in the last three months you felt less useful for patients?
- How has the intensity of your work changed with patients who are now later in the postpartum period?
- What unmet needs do you currently have as a patient navigator?
  - Can you tell me about a time a patient needed assistance that you could not provide her due to this deficiency?
- What lessons have you learned from your earlier patients that you’re applying now?
- What do you feel is the appropriate case load for a patient navigator?
  - Do you think this number would shift over time?

Do you have any other thoughts or stories you would like to share which you feel describe your role as a navigator or the benefits of patient navigation?
