# Supplementary material for: Identifying Health Systems Gaps as Perceived by Postpartum Patient Navigators at an Urban Academic Medical Center
Source: Womens Health Rep (New Rochelle). 2025 Jul 23;6(1):691–701. doi: 10.1177/26884844251362313 (PMC12413253; doi:10.1177/26884844251362313)
Supplement: Supplementary Data S1 [file 26884844251362313_supp_datas1.docx]

**RESPONSE TO REVIEWERS**

**Comments to the Author
Thank you for the reflections and revisions in response to the previous review. I have one clarification to the comment I made regarding assigning participant IDs to Table 3. While I understand the need for privacy, without some sort of differentiation it is difficult to know whether a theme/subtheme was generated from one experience or multiple. Given the small sample, I think it would be helpful to either differentiate these quotes or emphasize either in the paper itself or the table that the included quotes reflect multiple experiences collected in the course of the study.**

Thank you for the opportunity to provide additional clarification. In lines 142-146, we have explicitly stated that themes were only considered if identified by two or more navigators. Furthermore, we note that we corroborated navigator-identified themes with interviews from all participants to ensure they applied to the patient experience broadly, rather than individual participant narratives.
